# Early clinical efficacy analysis of enhanced recovery following surgery combined with interscalene brachial plexus block for arthroscopic rotator cuff repair

**DOI:** 10.1097/MD.0000000000035943

**Published:** 2023-11-10

**Authors:** Xiang Li, Hong-yang Jiang, Yong-jie Zhao, Si-zhuo Liu, Ling-xiao Pan

**Affiliations:** a Health Science Center, Ningbo University, Ningbo, China; b Department of Orthopedics and Sports Medicine, Li Huili Hospital Affiliated to Ningbo University, Ningbo, China.

**Keywords:** enhanced recovery after surgery, interscalene brachial plexus block, length of hospital stay, rotator cuff injury, VAS

## Abstract

To explore the early clinical value of enhanced recovery after surgery (ERAS) with interscalene brachial plexus block (ISB) for arthroscopic rotator cuff repair (ARCR). We enrolled 240 patients who underwent arthroscopic rotator cuff repair, randomly divided into 3 groups (n = 80 each). Groups A, B, and C underwent only surgery, surgery + ERAS, and ISB + surgery + ERAS, respectively. We analyzed the clinical data and postoperative indicators for the 3 patient groups. Group comparisons of clinical data and postoperative indicators revealed no significant differences in clinical characteristics (*P* > .05). Group C showed superior Visual Analog Scale scores at 0–6 and 6–24 hours postoperatively (*P* < .05), and the shortest length of hospital stay (LOS) (*P* < .05). At 6 weeks and 3 months postoperatively, Constant-Murley shoulder score and University of California-Los Angeles scores were better in Groups B and C than in Group A (*P* < .05). Joint swelling was more common in Group A than in Groups B and C (*P* < .05) but with no significant difference in the incidence of postoperative stiffness (*P* > .05). ERAS can relieve postoperative pain, shorten LOS, and help restore shoulder joint mobility, thereby reducing postoperative swelling. ISB + ERAS optimized pain control and allowed a shorter LOS, but had similar effects on early functional recovery and complications.

## 1. Introduction

The rotator cuff is composed of the supraspinatus, infraspinatus, subscapularis, and teres minor muscles, and is primarily responsible for rotational movements of the shoulder joint and maintaining shoulder stability. The prevalence of rotator cuff injuries can reach 30% to 50% in individuals over age 50.^[1]^ These injuries usually present with impaired mobility and pain, which can seriously affect patient quality of life. Arthroscopic rotator cuff repair (ARCR) is a minimally invasive operation that is recognized for its advantages, including minimal trauma, rapid recovery time, and good efficacy.^[2]^ ARCR is often associated with severe postoperative pain due to high pressure in the joint cavity and tension from the rivets.^[3]^ Currently, an increasing number of clinicians are applying the principles of enhanced recovery after surgery (ERAS),^[4–6]^ with the aim of reducing complications and shortening recovery time. One of the core measures of ERAS is pain management, defined as the maintenance of a postoperative Visual Analogue Scale (VAS) pain score controlled to <3.^[7]^

Interscalene brachial plexus block (ISB) provides effective analgesia by blocking conduction of painful stimuli, reducing dosage of intraoperative and postoperative analgesics, and consequently reducing the incidence of adverse effects, such as nausea and vomiting.^[8]^ In addition, ISB is more stable compared to general anesthesia (GA) with respect to intraoperative heart rate, blood pressure, and hemodynamics.^[9]^ This facilitates controlled lowering of blood pressure and improves clarity of the visual field.^[10]^ As the development of accurate visualization techniques has progressed, ultrasound guidance has improved the safety and accuracy of ISB by allowing accurate display of essential structures. As an element of ERAS, ISB plays an important role in reducing postoperative pain and improving recovery of daily activities of living, making an in-depth understanding of the role of ISB in ERAS essential.

Therefore, the present study was conducted to answer the following questions: How effective are ERAS with ISB and ERAS alone compared to conventional treatment for control of early postoperative pain? Are there differences in the length of hospital stay (LOS) and early functional outcomes? Are there differences in the incidence of postoperative complications? Answers to these questions will provide valuable evidence for future clinical treatments.

## 2. Materials and methods

### 2.1. Patient selection and general information

This study enrolled patients with rotor cuff repair, treated in our joint and sports medicine department between April 2022 and April 2023. The study was conducted in accordance with the Declaration of Helsinki and approved by the Institutional Review Board of Li Huili Hospital affiliated with Ningbo University (KY2023SL004-01).

The inclusion criteria were as follows: obvious shoulder symptoms with restricted motion and no improvement of symptoms after 2 months of conservative treatment; confirmation of rotator cuff tear on magnetic resonance imaging; first-time unilateral ARCR; American Society of Anesthesiologists classification I–II; and patients and family members being aware of the study treatment plan and providing written informed consent.

The exclusion criteria were as follows: history of shoulder fracture or prior surgical treatment; rotator cuff tear size >3 cm; presence of central or peripheral neurological diseases, such as epilepsy; and incomplete medical records or refusal to participate in the experimental study till completion.

In total, 240 patients met the above criteria. Patients were divided into Groups A, B, and C using the random number table method, with 80 cases in each group. Group A included 26 men and 54 women aged 57.60 ± 12.09 years (range: 22–78 years) treated with ARCR alone. Group B included 31 men and 49 women aged 56.85 ± 8.32 years (range: 35–70 years) and treated with ERAS + ARCR. Group C included 30 men and 50 women aged 55.90 ± 11.98 years (range: 26–80 years) and treated with ERAS + ISB + ARCR. No significant differences were observed in age, sex, body mass index, and duration of surgery among the 3 groups (all *P* > .05, Table 1).

**Table 1:** Patient demographics and clinical indicators (n, *x* ± *s*).

Variable	Group A	Group B	Group C	χ^2^/F	*P* value
Age (yr)	64.31 ± 7.25	65.68 ± 7.49	65.50 ± 7.07	0.831	.437
Sex (cases)					
Male	29	21	22	2.262	.369
Female	51	59	58		
BMI (kg/m^2^)	24.43 ± 3.40	23.44 ± 2.71	23.66 ± 3.02	2.326	0.100
Surgical duration (min)	81.89 ± 9.00	82.63 ± 7.46	82.56 ± 7.92	0.213	0.899

BMI = body mass index.

### 2.2. Treatment modalities

#### 2.2.1. Surgery.

All ARCR surgeries were performed by a senior chief physician. After achieving anesthesia, thorough disinfection, and draping, the affected limb was abducted and flexed in the anterior direction in preparation for suspension traction using the standard posterior approach for routine repair.

#### 2.2.2. Routine perioperative management.

Before the operation, patients were routinely instructed not to take pain medications, to fast for 8 hours, and to not drink water for 6 hours prior to surgery in order to ensure accuracy of the experimental study. The lavage fluid was maintained at room temperature, and the volume was not controlled. Interventions were performed for patients with blood pressure over 140/90 mm Hg. No drain placement was performed at the end of the procedure, and patients were allowed to eat food 6 hours postoperatively. After the operation, patients were placed in a shoulder brace and administered nonsteroidal anti-inflammatory drugs daily for pain management. Patients were instructed to perform functional rehabilitation exercises under the supervision of a rehabilitation physician.

#### 2.2.3. ERAS perioperative management.

Prior to the operation, the attending physician, anesthesiologist, rehabilitation physician, and the nurse-in-charge communicated with each patient in detail. This team comprehensively assessed physiological, psychological, mental, and nutritional status, and informed the patient about measures specific to ERAS, postoperative complications, and the rehabilitation plan. Based on the patient’s condition, targeted health education was provided, and an individualized ERAS plan was formulated. Patients were provided psychological counseling to alleviate adverse emotions, such as tension and anxiety. For patients with hypertension, short-acting antihypertensive drugs were administered on the day of surgery to control blood pressure. For patients with diabetes, blood glucose was strictly controlled. Patients with insomnia were treated with oral zolpidem tartrate tablets. Patients were instructed to avoid taking pain medication for as long as possible prior to surgery. During the operation, heating pads were used to maintain body temperature. The temperature of the operating room was controlled to 23°C, and the temperature of the irrigation fluid in the joint cavity was controlled to 36°C. The patient’s blood pressure was controlled and lowered as necessary, the levels of perioperative fluid input and perfusion were limited, and no drainage tubes were placed at the end of the operation. Patients were placed in the same shoulder brace as after operation, nonsteroidal anti-inflammatory drugs were administered for pain management, and hot and cold wet compresses were applied intermittently. Patients were instructed to perform the same functional rehabilitation exercises under the supervision of a rehabilitation physician. In addition, psychological guidance and distraction techniques were provided.

Although some of the perioperative interventions differed between groups, all postoperative rehabilitation measures were the same and were therefore treated as constants in the present study. In order to elucidate the effects of ERAS and ISB interventions, the remaining interventions had to be as consistent as possible to minimize errors in the study results. Other than the application of ISB, the perioperative management of patients in Group C was identical to that of Group B.

#### 2.2.4. Interscalene brachial plexus block.

ISB was performed under ultrasound guidance. After the puncture needle reached the target location under ultrasound guidance, 0.5 µg/kg dexmedetomidine compounded with 0.5% ropivacaine 15 to 20 mL was injected. The efficacy of the block was tested, and general anesthesia was induced after successful completion of the block.

### 2.3. Outcome indicators

The primary outcome was pain intensity, measured using the VAS score, measured at 0–6, 6–24, and 24–48 hours postoperatively. LOS, 1- and 3-month Constant-Murley shoulder scores (CMS), University of California-Los Angeles (UCLA) shoulder scores, and incidence of postoperative joint stiffness and swelling in the 3 groups were collected as secondary outcomes.

### 2.4. Statistical analysis

Data were analyzed using SPSS v. 25.0 (IBM, Armonk, NY). Continuous variables were expressed as the mean ± standard deviation and categorical variables were expressed as number of cases. After determining whether the data conformed to a normal distribution, normally distributed continuous variables were analyzed by one-way analysis of variance with post hoc tests. The Kruskal–Wallis test was performed for multiple sets of continuous variables not conforming to normal distribution. Categorical variables were compared using the chi-square test or Fisher’s exact test. *P* < .05 was considered statistically significant.

## 3. Results

### 3.1. Primary outcome

**Figure 1.**
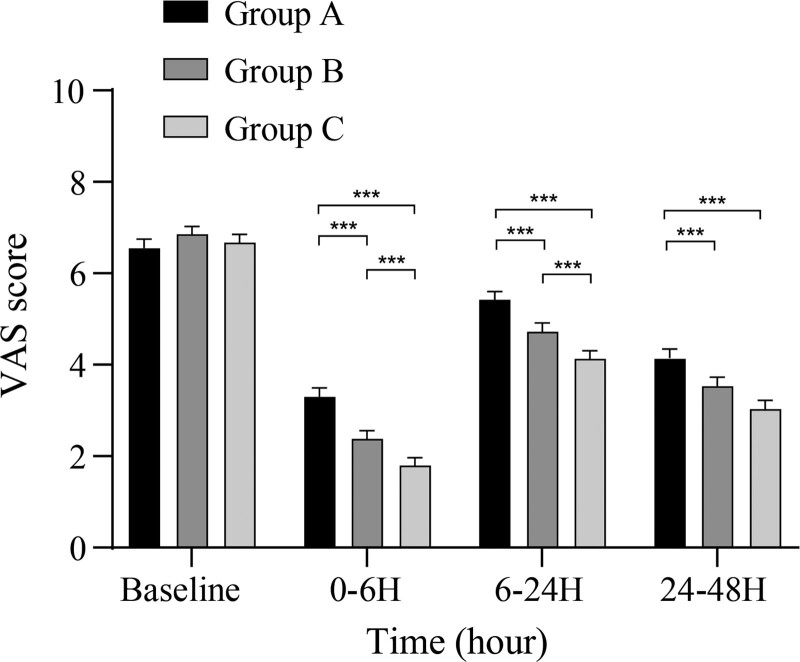
Comparison of VAS scores among the 3 groups at different time points. VAS = Visual Analog Scale.

VAS scores were compared as the primary outcome. Results at 0–6 and 6–24 hours postoperatively exhibited differences between the 3 groups (*P* < .05). Post hoc tests showed that Group C was superior to Group B (*P* < .05), which was in turn superior to Group A (*P* < .05). Results at 24–48 hours postoperatively showed that the VAS scores of Group A were significantly higher than those of Groups B and C (*P* < .05); however, the difference between Groups B and C was not significant (*P* > .05). The VAS scores are shown in Figure 1.

### 3.2. Secondary outcomes

#### 3.2.1. Length of hospital stay.

**Figure 2.**
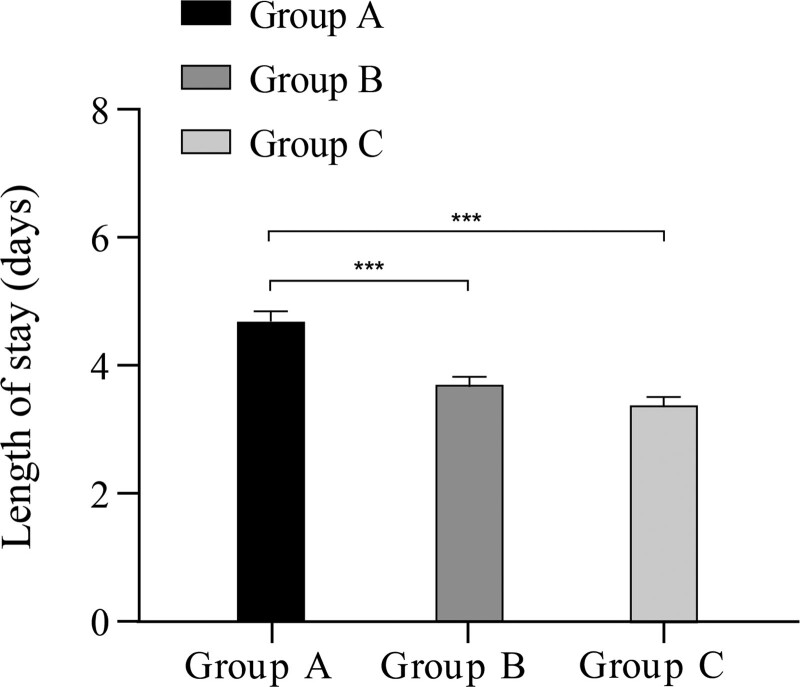
Comparison of LOS among the 3 groups. LOS = length of hospital stay.

A significant difference in the LOS was found among the 3 groups (*P* < .05). Post hoc tests showed a shorter LOS in Group C compared to Group B (*P* < .05), as well as in Group B compared to Group A (*P* < .05). (Fig. 2)

#### 3.2.2. CMS and UCLA scores.

**Figure 3.**
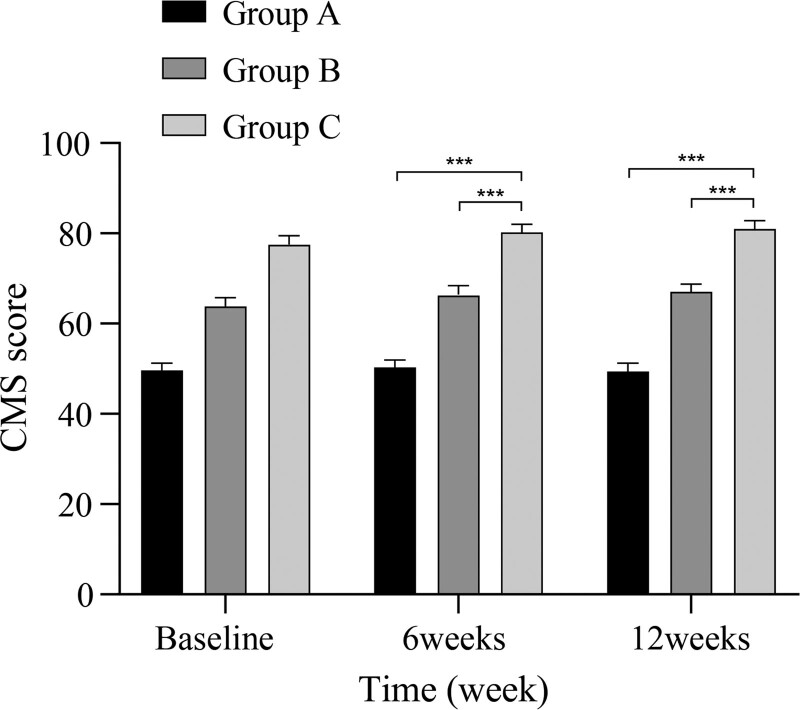
Comparison of CMS among the 3 groups at different time points. CMS = Constant-Murley shoulder score.

**Figure 4.**
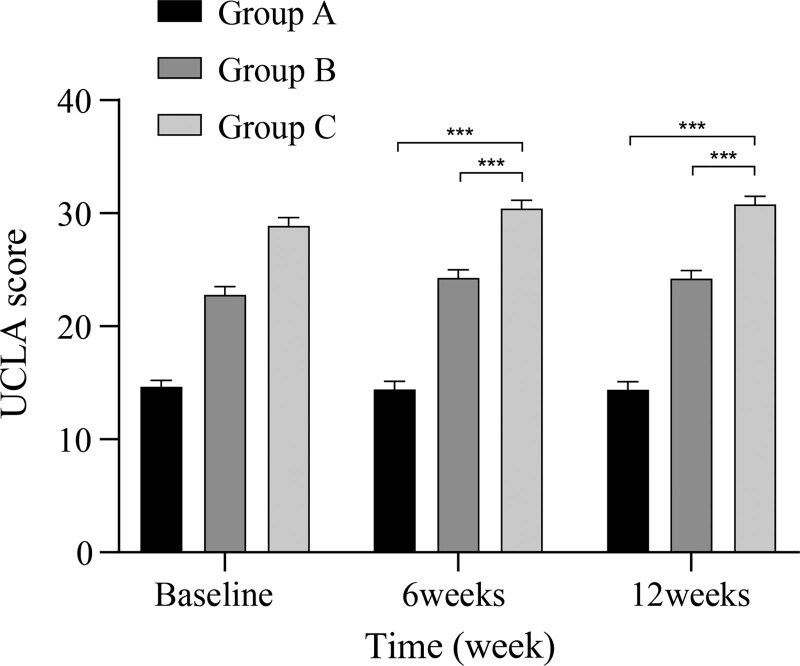
Comparison of UCLA scores among the 3 groups at different time points. UCLA = University of California-Los Angeles.

The preoperative shoulder function scores did not significantly differ among the 3 groups. However, at 6 weeks and 3 months postoperatively, the scores of Groups B and C were better than those of Group A (*P* < .05), even if the difference between the two was not significant (*P* > .05) (Figs. 3 and 4).

#### 3.2.3. Postoperative complications.

The incidence of postoperative stiffness did not significantly differ among the 3 groups (*P* > .05). Conversely, the incidence of joint swelling was significantly higher in Group A than in Groups B and C (*P* < .05) (Table 2).

**Table 2:** Comparison of incidence of postoperative complications among the 3 groups.

Variable	Group A, n (%)	Group B, n (%)	Group C, n (%)	χ^2^	*P* value
Joint stiffness	4 (5.00)	2 (2.50)	3 (3.75)	0.749	.911
Joint swelling	7 (8.75)	1 (1.25)	1 (1.25)	6.760	.022

## 4. Discussion

Shoulder surgery involves multiple muscles and ligaments. Patients undergoing ARCR experience more pain after surgery than those who undergo other shoulder arthroscopy procedures.^[11]^ In addition, the narrow space for arthroscopic surgery necessitates pressurizing the joint cavity for irrigation in order to obtain a clear view, resulting in significant pain caused by postoperative soft tissue swelling. In addition to affecting patient satisfaction, time to discharge, and psychological well-being, postoperative pain may influence recovery of joint function.^[12]^

The concept of perioperative ERAS was introduced by Danish surgeon Henrik Kehlet in 1990.^[13]^ ERAS scientifically optimizes and improves on the conventional set of perioperative measures through an evidence-based medical approach, including optimization of anesthesia, minimally invasive techniques, optimization of postoperative analgesic measures, and early rehabilitation guidance and exercise. This technique aims to minimize patients’ stress response, reduce surgery-related complications, shorten length of hospital stay, and achieve rapid recovery. ISB is simple to perform and is a common anesthetic method for ARCR.^[14]^ However, relatively few studies have investigated the outcomes of ISB combined with ERAS for postoperative pain management and early postoperative functional outcomes. Therefore, the present study aimed to investigate the effect of ISB combined with ERAS in the early postoperative period in patients undergoing ARCR.

Nerve block techniques modify the generation of responses to stress by modulating or inhibiting the transmission of noxious afferent signals during surgery.^[15]^ The present study showed that VAS scores at 0–6 and 6–24 hours postoperatively were significantly lower in group C (including patients who underwent ISB) than in groups A and B, suggesting that ERAS significantly reduces pain and discomfort in the early postoperative period and that ISB has an additional effect. At 24–48 hours postoperatively, groups B and C had better VAS scores than group A; however, the scores of groups B and C did not differ significantly. This suggests that the analgesic effect of ISB begins to diminish after 24 hours, whereas ERAS can still benefit the patient with sustained pain reduction. O’Donnell et al^[16]^ showed that VAS scores at 2 and 6 hours postoperatively in patients who underwent ISB were superior to those of patients who underwent GA. However, a prior meta-analysis by Kalthoff et al^[17]^ showed no significant difference in VAS scores at 24 and 48 hours postoperatively between patients who underwent ISB and GA, which agrees with our findings. The analgesic effect of a single-injection block is limited by factors, such as drug activity. Patients may experience a nociceptive hypersensitivity reaction, resulting in acute pain exacerbation within the first 48 hours.^[18]^ However, in the present study, we found no significant difference in VAS scores between groups B and C after operation. This may be due to the benefits of measures such as early activity, cold compression, and individualized psychological guidance in ERAS. Early activity and cold compression can reduce pain by improving blood circulation and reducing bruising and swelling at the wound. Furthermore, appropriate psychological interventions can boost patient confidence.

The results of the current study show that group A had the longest LOS whereas group C had the shortest LOS, which is consistent with previous findings.^[19,20]^ Among the steps involved in ERAS implementation, preoperative health education, intraoperative heat preservation, and early functional training can reduce the stress response to surgery and promote early recovery, which is essential for achieving early discharge.^[21]^ This result also confirms the feasibility and safety of ERAS optimization measures. ISB helps to alleviate postoperative pain and promotes recovery of voluntary movement, facilitating early discharge. However, administration of a nerve block has been shown to not significantly affect LOS compared to GA alone.^[22]^ We believe that this may be related to the insufficient sample size, which must be increased in future investigations.

Postoperative pain induces the stress response in the body and delays postoperative recovery, thus affecting functional recovery. However, Tirefort et al^[23]^ indicated that premature or excessive functional rehabilitation following treatment of rotator cuff injuries does not provide significant benefits. To the best of our knowledge, no studies have yet assessed the early functional outcomes of ERAS combined with ISB after undergoing ARCR.

The CMS score was adopted by the European Society for Surgery of the Shoulder and the Elbow as a standardized scale for the assessment of shoulder function and is now used worldwide.^[24]^ This score includes objective assessment indicators, such as shoulder range of motion and strength tests, as well as subjective assessment indicators, such as pain and daily activities. The UCLA score also includes metrics such as satisfaction. In the present study, we assessed shoulder functional outcomes by comparing postoperative CMS and UCLA scores in the 3 groups of patients. The results showed that both scores were higher in groups B and C than in group A at 6 weeks and 3 months postoperatively. Although group C scored slightly higher than group B, no significant difference was observed between the 2 groups. Strength of the rotator cuff muscles and stability of the shoulder joint could be gradually increased through implementing a scientifically validated postoperative ERAS rehabilitation protocol, consistent with the findings of Chen et al.^[25]^ Postoperative delays in rehabilitation progress due to fear of pain may account for the slightly lower scores in Group B than in Group C. However, our findings suggest that administration of ISB did not significantly impact early postoperative shoulder functional recovery.

Shoulder swelling and stiffness are common complications in the early postoperative period^[26]^; however, nerve block is known to reduce the incidence of shoulder surgery complications.^[27]^ In the present study, the incidence of postoperative swelling was significantly higher in Group A than in Groups B and C. Unlike the treatment-only group, intermittent cold compression was applied in Groups B and C, which helped to relieve swelling and pain; moreover, ISB was administered in Group C, which further facilitated postoperative pain relief. However, no significant difference was observed in postoperative stiffness between the 3 groups.

Prevention of shoulder stiffness requires early and standardized rehabilitation.^[28]^ We believe that the lack of difference between the effects of ERAS and ERAS with ISB administration was observed because all 3 groups of patients underwent postoperative rehabilitation and exercise.

This study has some limitations. Firstly, it had a small sample size and was conducted at a single center, which may have introduced bias. Further studies should be conducted at multiple centers in order to facilitate more scientific and accurate conclusions. Furthermore, the length of the follow-up period was short; the longest follow-up period was only 3 months. In the future, the follow-up period should be extended to facilitate observation of long-term efficacy.

## 5. Conclusion

We found that the combination of ERAS and ISB eliminates pain in the early postoperative period following ARCR, reduces LOS, and decreases the incidence of postoperative shoulder swelling. However, the effect on early postoperative functional recovery is similar.

## Acknowledgments

We would like to thank Editage (www.editage.cn) for English language editing.

## Author contributions

**Conceptualization:** Xiang Li, Hong-yang Jiang, Ling-xiao Pan.

**Data curation:** Xiang Li.

**Formal analysis:** Xiang Li, Yong-jie Zhao.

**Investigation:** Xiang Li, Ling-xiao Pan.

**Methodology:** Xiang Li, Ling-xiao Pan.

**Project administration:** Ling-xiao Pan.

**Software:** Xiang Li, Hong-yang Jiang.

**Supervision:** Ling-xiao Pan.

**Validation:** Xiang Li, Yong-jie Zhao, Si-zhuo Liu.

**Writing – original draft:** Xiang Li, Hong-yang Jiang, Si-zhuo Liu.

**Writing – review & editing:** Ling-xiao Pan.
